# Construction of a novel vector expressing the fusion suicide gene yCDglyTK and hTERT-shRNA and its antitumor effects

**DOI:** 10.3892/etm.2012.613

**Published:** 2012-06-18

**Authors:** JIA LI, GUIYING ZHANG, TING LIU, HUAN GU, LU YAN, BOLIN CHEN

**Affiliations:** Department of Gastroenterology, Xiangya Hospital, Central South University, Changsha, Hunan 410008, P.R. China

**Keywords:** RNA interference, suicide gene, gastric cancer, combined gene therapy, SGC7901

## Abstract

This study aimed to construct a novel recombinant expression vector, pcDNA3.1(-)hTERT-shRNA/yCDglyTK. Its bioactivity and antitumor effects were investigated in the SGC7901 human gastric cancer cell line. Interfering RNA (RNAi) targeting human telomerase reverse transcriptase (hTERT) was applied to construct the pYr1.1-hTERT-shRNA vector. The shRNA expression cassette (including U6 promoter) was subcloned into the pcDNA3.1(-) CV-yCDglyTK vector to build a new vector, pcDNA3.1(-) hTERT-shRNA/yCDglyTK, which was identified by restriction enzyme digestion and gene sequencing. All the plasmids were delivered into SGC7901 cells using calcium phosphate nanoparticles (CPNPs). Expression of yCDglyTK and hTERT was detected by immunofluorescence, real-time PCR and western blot analysis. MTT assays were applied to measure the cytotoxic effect of the plasmids with 5-fluorocytosine (5-FC). Cell apoptosis was detected by flow cytometry. Restriction enzyme digestion and gene sequencing confirmed that the recombinant vector pcDNA3.1(-)hTERT-shRNA/yCDglyTK had been successfully constructed. Immunofluorescence, real-time PCR and western blot analysis showed that yCDglyTK was expressed, and that hTERT expression was inhibited in cells transfected with the recombinant vector. The cells transfected with the recombinant vector were the most sensitive to 5-FC and the apoptosis rates of the cells were also increased. The pcDNA3.1(-)hTERTshRNA/yCDglyTK vector was constructed successfully; it was confirmed that targeting hTERT through RNAi could synergize with suicide gene therapy.

## Introduction

Gene therapy for cancer treatment is now gaining an increasing interest from across the globe (1,2). A variety of strategies have been used to date, including the inhibition of oncogene expression, tumor angiogenesis and multidrug-resistant gene expression, and the induction of tumor-suppressor and suicide gene expression or the induction of antitumor immunity. Combining these strategies in order to improve antitumor effects is plausible as carcinogenesis involves a multitude of factors, as well as multi-step and multi-stage processes (3).

Inducing a suicide gene to tumor cells holds broad applications in tumor treatment. This therapy involves introducing genes of non-mammalian enzymes into cancer cells, so that they can convert non-toxic prodrugs into highly toxic substances. This will, in turn, kill not only the infected cells but also the adjacent ones. Thymidine kinase (TK), which converts the prodrug ganciclovir (GCV) into phosphorylated GCV (p-GCV), and cytosine deaminase (CD), which converts the prodrug 5-fluorocytosine (5-FC) into 5-fluorouracil (5-FU), are two popular prodrug-converting non-mammalian enzymes and are encoded by the TK and CD genes, respectively. In the tumor cell, p-GVC inhibits cellular DNA synthesis, leading to tumor cell death via apoptotic/non-apoptotic mechanisms, whereas 5-FU, a chemotherapy drug, interferes with nucleoside metabolism by being incorporated into RNA and DNA, leading to cytotoxicity and cell death (4,5). Both have been incorporated into the two most common suicide gene/prodrug systems: the herpes simplex virus thymus kinase (HSV-TK) gene/GCV system (HSV-TK/GCV) and the *E. coli* cytosine deaminase (Ec-CD) gene/5-fluorocytosine (5-FC) system (Ec-CD/5-FC) (6,7). A combination of two or more suicide genes, or the combination of the suicide gene with other genes to make a new fusion gene, would theoretically confer various synergistic effects, as a single suicide gene or a single gene interfering therapy easily leads to drug resistance, and its treatment effects vary according to tumor cell type.

Previous studies have shown that a suicide gene, combined with chemotherapy drugs or other genes, is able to enhance antitumor action (8–10). The fusion gene that connects the CD gene with the TK gene is a widely used one. With the prodrug-converting enzyme activities of CD and TK genes, it breaks the dependence of tumor cell types, eliminates drug resistance and expands the application of the therapy.

Hamstra *et al* (11) found that the CD gene from yeast (yCD) in comparison with the *E. coli* CD gene (bCD) can more effectively alter catalytic 5-FC into 5-FU. In our previous study, we constructed the fusion suicide gene yCDglyTK containing yeast CD, using enhanced the CEA promoter to drive its expression in carcinoembryonic antigen (CEA)-positive cells. We found that this fusion suicide gene was more effective on the SGC7901 human gastric adenocarcinoma cell line when used with the prodrug 5-FC alone (12).

Telomerase, a ribonucleoprotein enzyme responsible for adding the telomeric repeats onto a chromosome, plays an important role in the development of cellular immortality and oncogenesis (13,14). Previous studies have shown that telomerase activity is found in 85–90% of all human tumors, but not in their adjacent normal cells (15,16). This makes telomerase a good target not only for cancer diagnosis, but also for the development of novel therapeutic agents (17,18).

Human telomerase is composed of three components: human telomerase RNA (hTR), telomerase reverse transcriptase (hTERT) and telomerase associated protein 1 (hTEP1). hTERT is the catalytic subunit of telomerase. It is expressed in cells with telomerase activity and its expression level is positively correlated with telomerase activity (19). The SGC7901 human gastric adenocarcinoma cell line is the major subtype of gastric cancer cell lines with high hTERT gene expression (20). RNA interference (RNAi) targeting hTERT reduces the expression of the mRNA and protein of hTERT, exerting antitumor effects.

In our previous studies, a plasmid carrying the fusion suicide gene yCDglyTK was constructed (12,21). In order to enhance the antitumor effect of the system, in the present study, this fusion suicide gene was combined with hTERT-targeted shRNA, and a new combined plasmid pcDNA3.1(-) hTERT-shRNA/yCDglyTK was constructed. Its bioactivities and antitumor effect were investigated in the SGC7901 human gastric cancer cell line.

## Materials and methods

### Cell line

The SGC7901 human gastric cancer cell line was obtained from the Central Laboratory of Xiangya Hospital, Central South University (Changsha, China). SGC7901 cells were grown in RPMI-1640 containing 10% calf serum at 37°C in a 5% CO_2_ humidified incubator.

### Reagents

Restriction enzymes *Xho*I, *Nhe*I, *Eco*RI, *Xba*I, *Hin*dIII were purchased from MBI Fermentas. T4-DNA ligase (New England Biolabs), rTaq DNA polymerase (Takara), SYBR-Green Real Master mix, DNA Marker IV and DNA Marker DL2000 (Tiangen Biotech Co.) were used. Calf serum, RPMI-1640 (Thermo Scientific), trypsin (Beyotime). G418 (Sigma), calcium phosphate nanoparticles (from our laboratory, Xiangya Hospital, Changsha, China) were obtained. Reverse transcriptase kit (MBI Fermentas), goat anti-CD antibody (Abcam), mouse anti-human telomerase reverse transcriptase antibody (Santa Cruz Biotechnology, Inc.), the cell cycle detection kit (KeyGen Biotech Co., Ltd.) and 5-fluorocytosine (5-FC; Sigma) were also used in the study.

### Plasmids

Plasmids used in this study are listed in Table I. The cells were set according to the following groups: i) SGC7901 (group A; control), ii) SGC7901/Null (group B; sham), iii) SGC7901/TERT-siRNA (group C; pTERT), iv) SGC7901/yCDglyTK (group D; pCD/TK) and v) SGC7901/TERT-siRNA-yCDglyTK (group E; pTERT/CD/TK).

### Construction of shRNA-directed hTERT-expressing plasmid

Following a searching for the hTERT mRNA sequence in GenBank, according to RNAi design software (Integrated DNA Technologies, Inc., Coralville, IA, USA) and Qian *et al* (22), we selected 5′-TGGTGGATGATTTCTTGTT-3′ as the target sequence. We designed a pair of complementary short hairpin RNA (shRNA). Oligonucleotides were chemically synthesized by Shanghai Health Industry. The sequences were as follows: hTERT-shRNA, F, 5′-CACCTGGTGGATGATTTCTTGTTTTCAAGACGAACAAGAAATCATCCACCATTTTTTG-3′ and R, 5′-AGCTCAAAAAATGGTGGATGATTTCTTGTTCGTCTTGAAAACAAGAAATCATCCACCA-3′. The shRNA template oligonucleotides were cloned to pYr1.1 between the *Xho*I and *Eco*RI restriction sites. The expression of shRNA was regulated by the U6 promoter. Then we sequenced the interfering plasmid pYr1.1-hTERT-shRNA for confirmation of the target sequence.

### Construction of a new plasmid pcDNA3.1(-)hTERT-shRNA-yCDglyTK

shRNA expression cassette of pYr1.1-hTERT-shRNA was amplified by PCR (containing the U6 promoter). Primer sequences were as follows: P1, 5′-GCTAGCATCCAAGGTCGGGCAGGA-3′ and P2, 5′-TCTAGAGGTCTCGAGCTCAAAAAATGGT-3′, product was 356 bp. The conditions of PCR were P1 (10 μM) 0.25 μl, P2 (10 μM) 0.25 μl, ddH2O 19.75 μl, 10X LA PCR buffer (Mg^2+^ Plus) 2.5 μl, DNTPs (2.5 mM) 1 μl, LA Taq polymerase 0.25 μl and template 1 μl. The thermal cycle profile for PCR was 94°C for 5 min, followed by 30 cycles of 20 sec at 94°C, 25 sec at an annealing temperature of 53°C, 25 sec at 72°C, and an additional 3 min of incubation at 72°C after completion of the last cycle for extension.

PCR products were analyzed using agarose gel electrophoresis and stored at 4°C. The plasmid pcDNA3.1(-)CV-yCDglyTK was digested by *Nhe*I and *Xba*I. The digestion products were analyzed in agarose gel electrophoresis. Then, the hTERT-shRNA expression cassette and the target plasmid pcDNA3.1(-) CV-yCDglyTK linear fragments were recovered, and the two fragments were connected to form the plasmid pcDNA3.1(-) hTERT-shRNA-yCDglyTK. The new plasmid was transformed into competent *E. coli* DH 5α, then colonies were picked and plasmids were extracted. We subsequently sequenced the new plasmid and confirmed that the sequence was correct.

### Establishment of stably transfected cell lines

SGC7901 cells were plated in four 6-well plates at a density estimated to reach 80% confluence after 24 h. Transfection was performed using calcium phosphate nanoparticles. Calcium phosphate nanoparticles were respectively added to the plasmid pYr1.1 blank, pYr1.1-hTERT-shRNA, pcDNA3.1(-)CV-yCDglyTK and pcDNA3.1(-)hTERT-shRNA/yCDglyTK. SGC7901 cells were then transfected with each of the plasmid transfection mixtures. To select the SGC7901 cells which stably expressed the plasmids, the cells were treated with 400 μg/ml G418 for 14 days until all the non-transfected control cells were killed. The cells continued to be cultured with 200 μg/ml G418, and the medium was replaced every 3 days during the course. At the end of the culture period, the cells of the different colonies were picked and cultured for 3 weeks. The stably transfected cell lines were then established for the subsequent studies.

### Immunofluorescence assay

All of the SGC7901 cells (transfected and non-transfected) were plated in 6-well plates until they reached 60% confluence. The plates were washed with phosphate-buffered saline (PBS), treated with 4% paraformaldehyde for 20 min, permeabilized with 0.3% Triton for 15 min, and blocked with 1% BSA for 30 min, and the primary antibodies (mouse anti-human telomerase reverse transcriptase antibody and goat anti-CD antibody) were added. Incubation was carried out in a wet box at 4°C overnight. The plates were washed in PBS again, adding secondary antibodies (respectively Cy3-labeled anti-mouse and FITC-labeled anti-goat), and incubation was carried out at 37°C for 1 h, followed by washing with PBS. Cells were dehydrated and mounted with antifade mounting media, and observed and photographed under a fluorescence microscope against time.

### Real-time quantitative PCR

The gene mRNA level was analyzed using real-time quantitative PCR (RT-qPCR). First, the four SGC7901 cell groups were collected. Total RNA from the cells was extracted using a TRIzol reagent. The concentration of RNA was determined by measuring the absorbance at 260 nm using a RNA spectrophotometer (PerkinElmer, Fremont, USA); the cDNA was synthesized using a reverse transcriptase kit (MBI Fermentas, Hanover, MD, USA). The reaction conditions were as follows: cDNA 1 μl, sense 2 μl, antisense 2 μl, 2X SYBR-Green qPCR mix 25 μl and ultra-pure water to make up 50 μl. The thermal cycle profile for PCR was 95°C for 3 min, followed by 40 cycles of 30 sec at 95°C, and 40 sec at a annealing temperature of 60°C. The PCR instrument detected the fluorescence signal. After the reaction, 2 μl of product were used in 1.2% agarose gel electrophoresis.

The following specific primers were used: hTERT primers (expected fragment size 195 bp), sense, 5′-ACACCTGCCGTCTTCACTTC-3′ and antisense, 5′-TAGGGTCCTTCTCAGGGTCT-3′; yCDglyTK primers (expected fragment size 240 bp), sense, 5′-GGTGTTCCTATTGGCGGATGTCT-3′ and antisense, 5′-ACCGACAACACAGCGTGGAAT-3′; β-actin as internal reference (size 254 bp), sense, 5′-CTGTCTGGCGGCACCACCAT-3′ and antisense, 5′-GCAACTAAGTCATACTCCGC-3′.

### Western blot analysis

SGC7901 cells of the different groups were harvested, washed with PBS, and the cell lysate was cracked for 30 min on ice. The product was centrifuged at 12,000 rpm for 15 min; the supernatant was extracted for determination of the total protein. The protein concentration was detected by the BCA method; 40 μg protein and the corresponding volume of 6X loading buffer were diluted, and the mixture was boiled at 100°C for 5 min. The protein samples were electrophoresed in 15% SDS-PAGE. The gel contents were electrotransferred to PVDF membranes, and the membranes were blocked in 5% skimmed milk for 2 h at room temperature. Subsequently, the membranes were incubated with primary antibodies (mouse anti-human telomerase reverse transcriptase antibody and goat anti-CD antibody) at 4°C overnight, and with secondary antibodies (anti-mouse and anti-goat) at room temperature for 2 h, with three washes after incubations. The immunolabeled proteins were detected by enhanced chemiluminescence (ECL). An endogenous housekeeping gene and β-actin were also quantified and used to normalize hTERT and CD.

### Detection of 5-FC sensitivity of the cells

SGC7901 cells of all the groups were plated in 96-well plates, and cultured at 37°C in a 5% CO_2_ cell incubator, with the medium replaced daily. Untransfected SGC7901 cells were set as the control group; the other 4 groups were cultured with 200 μg/ml 5-FC. After a 96-h culture, 20 μl of MTT (5 mg/ml) was added and incubated for 4 h. The supernatant was absorbed carefully; 150 μl DMSO was added to each well. The cells were kept away from light for 10 min at room temperature. The absorbance was measured at 570 nm wavelength (OD570) with a model 680 microtiter plate reader (Bio-Rad, USA). Experiments were repeated 3 times and each group was set with 5 parallel wells.

### Detection of the apoptosis rate in the cells

SGC7901 cells in each group were collected, washed with phosphate-buffered saline (PBS), and then mixed with 70% ethanol at 4°C overnight. After being washed with PBS, 100 μl of RNase A was added to the cells at 37°C for 30 min, then lucifuged with 400 μl PI at 4°C for 30 min. Flow cytometry (Beckman Coulter, Inc., USA) was used for analyzing the cells. The assays were repeated three times.

### Statistical analysis

All data are expressed as the mean ± standard deviation (SD). Statistical analysis was performed by SPSS 13.0 and significance was defined as P<0.05.

## Results

### Construction and confirmation of the plasmid pcDNA3.1(-) hTERT-shRNA/yCDglyTK

**Figure 1:**
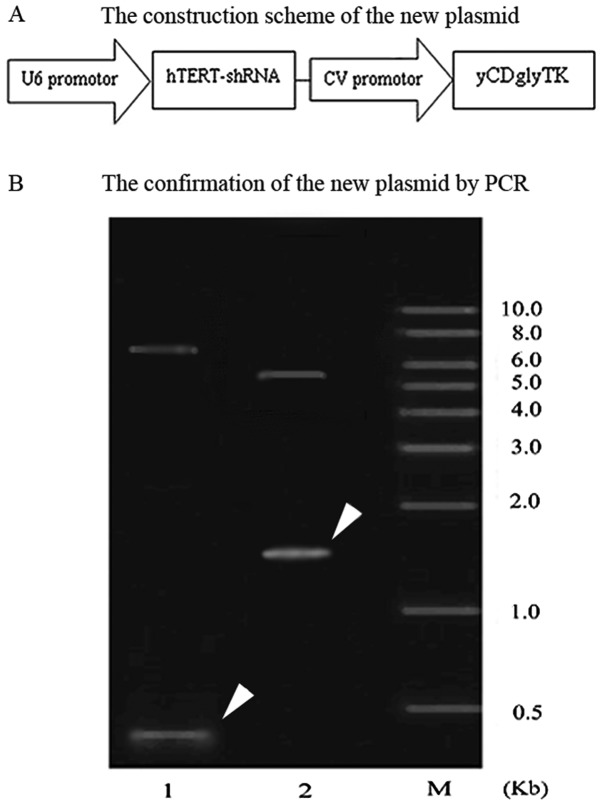
Construction scheme and confirmation of plasmid pcDNA3.1(-) hTERT-shRNA/yCDglyTK. (A) Construction of the novel plasmid. (B) Confirmation of plasmid pcDNA3.1(-)hTERT-shRNA/yCDglyTK. PCR analysis shows the amplified fragment of hU6-hTERT-shRNA expression cassette (lane 1, lower band) and fusion suicide gene yCDglyTK (lane 2, lower band). Arrows indicate the amplified fragments of the introduced shRNA and suicide gene. Lane 1, pcDNA3.1(-)hTERT-shRNA/yCDglyTK digested by *Nhe*I+*Xha*I; lane 2, pcDNA3.1(-)hTERT-shRNA/yCDglyTK digested by *Xho*I+*Hin*dIII; lane M, kb ladder.

The construction scheme of the expression plasmid pcDNA3.1(-)hTERT-shRNA/yCDglyTK is shown in Fig. 1A. The shRNA expression cassette was amplified by PCR using pYr1.1-hTERT-shRNA as a template. hTERT-shRNA expression cassettes were subcloned to pcDNA3.1(-)-yCDglyTK, thus constructing the new plasmid pcDNA3.1(-)hTERT-shRNA/yCDglyTK. The new plasmid was extracted and confirmed by PCR (Fig. 1B). The shRNA is shown as a band of ∼356 bp in lane 1, and yCDglyTK as a 1654-bp band in lane 2, confirming that both the exogenous hTERT interfering shRNA and the fusion suicide gene yCDglyTK were successfully inserted into the plasmid.

### Establishment of stably transfected cell lines

SGC7901 cells were cultured with G418 following the procedure stated above. After 7 days, the cells in the control groups were killed. Three weeks later, clones were formed in the transfected groups. The cells were continually cultured with G418 culture, and at the end of the culture, only the cells that had been successfully transfected with the plasmids survived. As a result, stably transfected cell lines were established and were further analyzed in the following experiments.

### Immunofluorescence assay detecting gene expression

**Figure 2:**
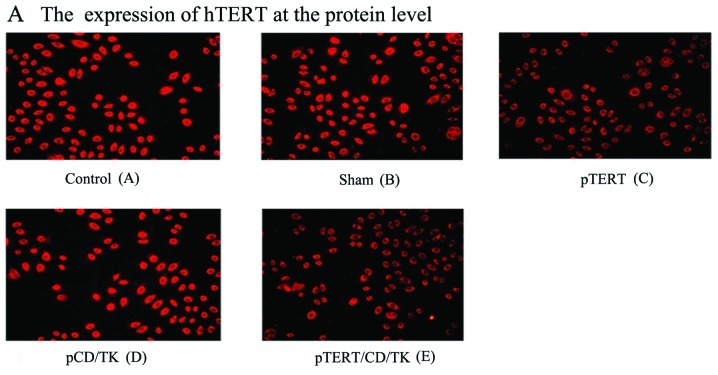

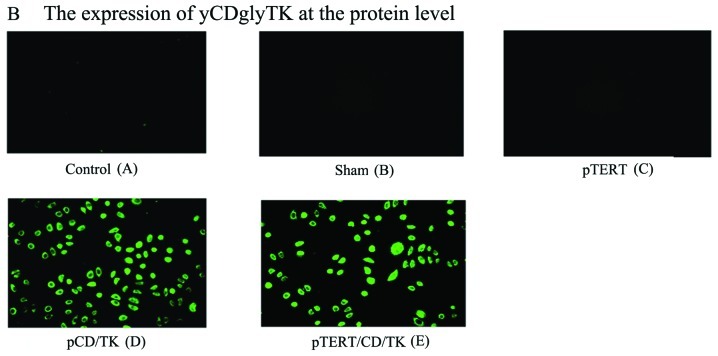
Representative image of immunofluorescence assay of the cells. (A) Fluorescence signals in the cells of groups C and E were obviously weaker compared with the other groups, indicating that the hTERT expression was downregulated by hTERT-shRNA. (B) Positive signals were only observed in the cells of groups D and E, indicating that the newly constructed fusion suicide gene CD/TK was successfully inserted into the cells of groups D and E (magnification, ×20).

Immunofluorescence detection showed that the expression of hTERT in the cells of groups C (pTERT) and E (pTERT/CD/TK) was significantly weaker than in groups A (control), B (sham) and D (pCD/TK) (Fig. 2A). Fusion suicide gene CD/TK protein was detected in the cells of groups D and E; there was no CD/TK expression in the other groups (Fig. 2B). This demonstrated that the expression of hTERT in groups C and E was inhibited, and that CD/TK protein was expressed in groups D and E. The plasmid pcDNA3.1(-)hTERT-shRNA/yCDglyTK regulated the shRNA hTERT and CD/TK expression.

### RT-qPCR and western blot analysis detecting gene expression

**Figure 3:**
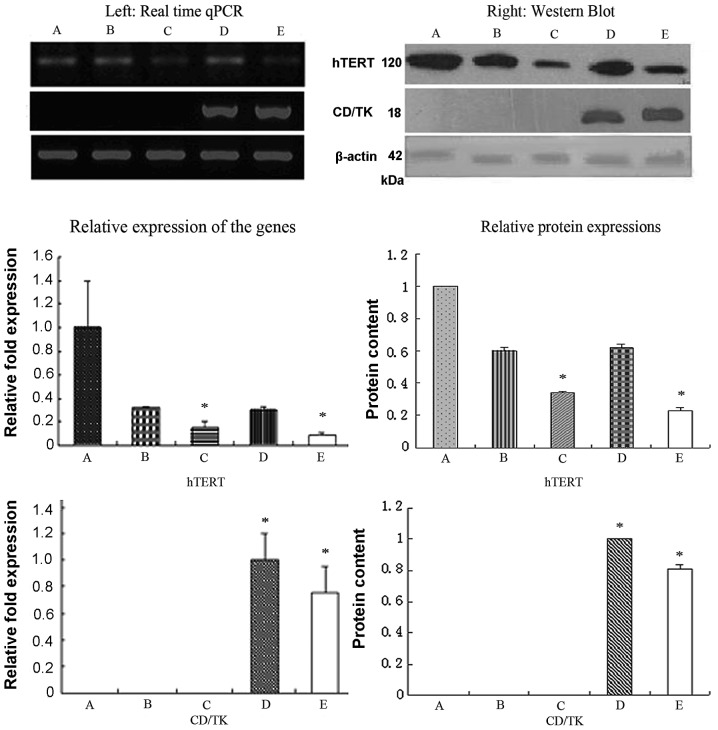
Expression of hTERT and yCDglyTK detected by RT-qPCR and western blot analysis. Both RT-qPCR and western blot analysis showed a similar change that was consistent with the findings of the immunofluorescence assay. Left,RT-qPCR shows the profiles of hTERT and CD/TK mRNA. Right, western blot analysis shows the expression of hTERT and yCDglyTK proteins. β-actin was used as a loading control. ^*^P<0.01 vs. all other groups.

RT-qPCR and western blot analysis (Fig. 3) showed that the hTERT expression at both the mRNA and protein levels was significantly lower in groups C and E (P<0.01), compared with groups A, B and D. There were no significant differences between groups C and E (P<0.01). These results suggest that pYr1.1-hTERT-shRNA and pcDNA3.1(-)hTERT-shRNA-yCDglyTK inhibited the expression of hTERT. The expression of fusion suicide gene-CD/TK was also detected by RT-qPCR and western blot analysis; in groups E and D, the CD/TK gene was expressed at both the mRNA and protein level, whereas no expression was observed in the other groups. This suggests that in the new combined gene plasmid pcDNA3.1(-)hTERT-shRNA/yCDglyTK, the U6 promoter regulates the shRNA hTERT expression and enhances the CEA promoter in regulating CD/TK expression.

### 5-FC sensitivity in the cells

**Figure 4:**
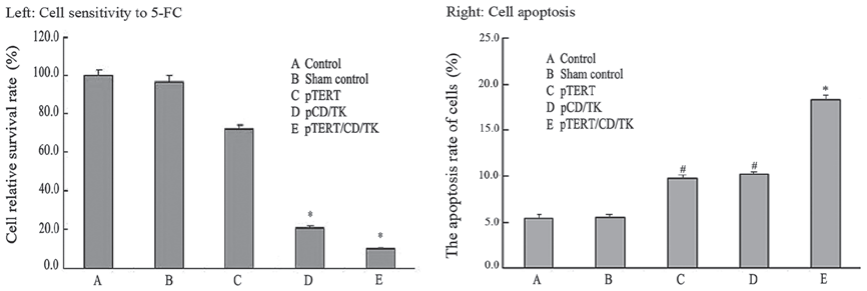
Sensitivity to 5-FC and the apoptosis rate of the cells. Data from MTT analysis (left) showed that the relative cell survival rates were significantly lower in groups D and E, although there was a decrease of the rate in group C; no significance was found in comparison with groups A and B. Data from flow cytometry (right) showed that apoptosis was increased in groups C–E, as cells in these groups were successfully transfected by the plasmids. ^*^P<0.01 vs. all other groups. ^#^P<0.01 vs. groups A, B and E.

The survival rate of the control group was 100%. With prodrug 5-FC treatment for 96 h, the relative survival rate of the control group was not significantly decreased, suggesting that the cell toxicity of 5-FC is limited. The relative survival rate of group C slightly decreased, suggesting that hTERT expression inhibited cell proliferation. The cells of groups D and E were sensitive to 5-FC; their relative survival rates were significantly lower than that in the control groups (A and B) and group C, with group E experiencing the greatest decline in survival rate (P<0.05) (Fig. 4). This suggests that the newly constructed plasmid pcDNA3.1(-) hTERT-shRNA/yCDglyTK inhibits cell proliferation more effectively.

### Detection of the apoptosis rate of the cells in each group

The apoptosis rate of each cell group was measured by flow cytometry (Fig. 4). The results showed that the apoptosis rates of groups A and B were 5.45±0.45 and 5.58±0.38%, respectively. The apoptosis rates of groups C, D and E were 9.76±1.54, 10.34±1.27 and 18.38±1.29% respectively, which were statistically higher in comparison with the control groups (P<0.01), indicating that the new plasmid pcDNA3.1(-)hTERT-shRNA/yCDglyTK induced cell apoptosis more effectively.

## Discussion

In gene therapy for cancer treatment, the key factors for success lay in finding effective combinations of genes and efficiently delivering them into tumor cells to regulate multiple gene expression, since carcinogenesis is a multi-step and multi-stage process (3). In this study, a novel plasmid, pcDNA3.1(-)hTERT-shRNA/yCDglyTK, was constructed successfully and delivered into SGC7901 cells by calcium phosphate nanoparticles. This new plasmid consists of a fusion suicide gene yCDglyTK and a hTERT/shRNA gene. Through comparisons of all the cell groups (including normal and sham control, single and multiple gene groups), we found that SGC7901 cells, which had been transfected with the plasmid carrying both hTERT/shRNA and the fusion suicide gene, were the more sensitive to the prodrug 5-FC. In these cells, the apoptosis rate was increased and the cell survival rate was decreased as a result of successful transfection of the new plasmid. The cell survival rate was slightly decreased in the sham control group, suggesting that the prodrug 5-FC cytotoxicity in normal gastric cancer was limited.

The fusion suicide gene, yCDglyTK, was constructed in our previous study (21), where it was expected to work with two prodrugs, 5-FC and GCV. However, in that study, we found that this fusion suicide gene demonstrated a stronger antitumor effect only when used with 5-FC. One explanation is that the TK/GCV system may reduce the cytotoxicity of the CD/5-FC system (23), and this was the reason why we used 5-FC alone in this study.

As hTERT plays an important role in the development of cellular immortality and oncogenesis, the inhibition of hTERT expression would be an ideal technique for gene therapy. Many studies have shown that hTERT gene expression in gastric cancer is increased, the tumor cell growth is suppressed, and tumor cell apoptosis is induced by inhibiting hTERT expression using the RNAi technique (19,20).

In the present study, we combined hTERT-specific RNAi with the fusion suicide gene to obtain a more effective antitumor effect. We first built the interference plasmid pYr1.1-hTERT-shRNA targeting the hTERT gene. Then, the shRNA expression cassette (including the U6 promoter) was subcloned into pcDNA3.1(-) CV-yCDglyTK to construct the combined gene plasmid. In this plasmid, there were two promoters regulating three different genes: U6 promoter regulating hTERT-shRNA expression, and the enhanced CEA promoter regulating the expression of the fusion suicide gene yCDglyTK. This new plasmid was delivered into SGC7901 cells and was verified by the establishment of the stably transfected cell line in G418 culture, by immunofluorescence assay, RT-qPCR and western blot analysis. Strong yCDglyTK expression in SGC7901 cells transfected with this new plasmid was noted, whereas hTERT expression was significantly downregulated in those cells.

In conclusion, the plasmid pcDNA3.1(-)hTERT-shRNA/yCDglyTK, which consists of an hTERT gene-specific shRNA and a fusion suicide gene yCDglyTK, was successfully constructed in this study. It was confirmed to have a synergistic antitumor effect on the SGC7901 human gastric cancer cell line.

## Figures and Tables

**Table I:** Plasmids used in this study.

Plasmids	Antibiotic resistance	Characteristics of the insert	Source
pYr1.1	Kan/Neo	hU6 promoter, blank	Yrbio, Changsha, China
pcDNA3.1(-)CV-yCDglyTK	Amp/Neo	CEA promoter, yCDglyTK gene	Constructed in our laboratory
pYr1.1-hTERT-shRNA	Kan/Neo	hU6 promoter, hTERT-shRNA	Constructed in our laboratory
pcDNA3.1(-)hTERT-yCDglyTK	Amp/Neo	hU6 promoter, hTERT-shRNA, CEA promoter, yCDglyTK gene	Constructed in our laboratory
